# Pharmacological and non-pharmacological interventions in management of peripheral venipuncture-related pain: a randomized clinical trial

**DOI:** 10.1186/s12887-023-03855-z

**Published:** 2023-02-03

**Authors:** Zhuowen Yu, Yiwen Zhou, Xiaofeng Xu, Lili Lin, Qian Le, Ying Gu

**Affiliations:** Children’s Hospital of Fudan University, No. 399, Wanyuan Rd, Minhang District, Shanghai, China

**Keywords:** Venipuncture pain, Distraction, EMLA emulsion, Children

## Abstract

**Background:**

Venipuncture is a routine nursing procedure in the pediatric ward for blood collection and transfusion. However, this procedure can cause severe pain and distress if not adequately managed.

**Methods:**

Children aged 3–16 years old were randomized into three groups: EMLA group, distraction group, and combined group. The primary outcome was children’s self-reported pain scored using the Wong-Baker FACES® Pain Rating Scale. The parents-reported and observer-reported pain were scored using the Revised Face, Legs, Activity, Cry and Consolability Scale, and children’s salivary cortisol levels, heart rate, percutaneous oxygen saturation, venipuncture duration and retaining time of IV cannulas were the secondary outcomes.

**Results:**

A total of 299 children (167 male, 55.8%, median age 8.5) were enrolled: EMLA group (*n* = 103), distraction group(*n* = 96) and combined group(*n* = 100). There was no statistical difference in self-reported pain (*P* = 0.051), parent-reported pain (*P* = 0.072), and observer-reported pain (*P* = 0.906) among the three groups. All three interventions can decrease children’s pain during IV cannulations. Additionally, the distraction group's salivary cortisol levels were lower than the combined group(*P* = 0.013). Furthermore, no significant difference was observed in the heart rate(*P* = 0.844), percutaneous oxygen saturation (*P* = 0.438), venipuncture duration (*p* = 0.440) and retaining time of IV cannulas (*p* = 0.843) among the three groups.

**Conclusions:**

All three groups responded with slight pain during the peripheral venipuncture procedure. Therefore, medical workers in pediatric settings can use the interventions appropriate for their medical resources and availability while involving parents and children’s preferences whenever possible.

**Trial registration:**

This trial was registered on https://register.clinicaltrials.gov/ (Gov.ID NCT04275336).

## Background

Patients in the pediatricsetting often experience invasive procedures like blood collection, immunization and intravenous (IV) catheterization, which bring about pain, stress and fear during their treatment [1, 2]. Failure to manage children’s pain and distress during needle procedures can lead to anxiety at follow-up and significantneedle phobias persisting into adulthood [3, 4]. Studies have shown that pain can be severe during venipuncture without interventions. Dalvandi and Sikorova et al. reported that VAS pain scores of children reach 7 (severe pain) and CHEOPS pain scores of children reach 9 (severe pain) if there are no analgesic interventions during venipuncture [5, 6].

Generally, pain management can be categorized into pharmacological and nonpharmacological interventions. Local anesthetics is one of the main pharmacological interventions in needle-related pain management, which can penetrate the cuticle and epidermal layer of intact skin, enter the dermis where nerve endings are located and relieve pain [6]. A eutectic mixture of local anesthetics (EMLA) emulsion containing 25 mg lidocaine and 25 mg prilocaine per gram has been studied in pediatric settings to manage venipuncture pain since it is effective and less invasive [7, 8]. A meta-analysis of 20 studies conducted to determine the effectiveness of EMLA on venipuncture shows a significant reduction of pain perception in 85% of patients [9].

However, it has been reported that the pain scores can reach up to 5 points (moderate pain) when local anesthesia is applied [10]. Some non-pharmacological strategies have been studied for needle procedures in children, such as distraction techniques, cognitive and behavioral therapy, hypnosis and memory alteration [11]. Among those interventions, distraction is the simplest, can be applied immediately and requires minimal prior training [11]. A systematic review showed that distraction could effectively alleviate pain from needle-related operations [12]. In related research, 5 min of virtual reality intervention positively impacted pain perception [13, 14].

There are limited data studies on merging pharmacological and non-pharmacological interventions to compensate for each other’s inadequacies. Hence, this study aimed to evaluate the effect of combining topical anesthetic and distraction techniques in comparison with the intervention applied singly. As a result, we expect a better effect from integrating pharmacological and non-pharmacological interventions.

## Methods

### Study design and setting and participants

A three-arm parallel, randomized controlled trial was conducted at the Children's Hospital of Fudan University on February 19. 2020 to August 10. 2020 in Shanghai, China. The study protocol was registered on https://register.clinicaltrials.gov/ as the number of Gov.ID NCT04275336 on February 19, 2020. Children who were scheduled to undergo IV cannula insertion in the Respiratory and Gastroenterology ward were included in this study. Primary eligibility criteria included children aged 3–16 years who were to receive their first peripheral IV puncture during hospitalization. The exclusion criteria are as follows: Children who 1) were not in their first hospitalization; 2) couldn't understand the pain rating scale; 3) had a hearing or vision impairment; and 4) declined to participate 5) didn't need IV cannulations during the hospitalization.

### Randomization and blinding

Simple random sampling was applied in this study. Eligible participants were randomly assigned to three groups via a computer-generated sequence. Opaque and sealed envelopes were sequentially numbered. The ward nurse, who was blinded to this study, assigned the intervention using the next randomization envelope. Children and their parents were informed to which group they had been assigned.

The observer rated the children’s pain by watching the real-time surveillance video when the nurse performed cannulation. Only a child’s face and body, including the puncture site shown in the camera’s viewfinder to ensure the observer was blinded. The observer in this study is a senior pediatric nurse of the surgical department, who has received training in pain assessment and didn’t participate in research design and statistics and was ignorant of all the study interventions. The statisticians remained blinded until the final analysis. The child and parents couldn’t be blinded in this study.

### Sample size

The sample size calculation formula of the quantitative experiment with superior efficacy was used, α = 0.05, degree of certainty (1-β) = 0.8, and Wong-Baker FACES® Pain Rating Scale(WB) scores were used as the primary outcome. According to the score of the WB of the EMLA group, distraction group, and combined group in the pre-test, the maximum difference was 1.5, the standard deviation was 3, and the sample size was calculated as 100 people in each group, with a total of 300 samples required, with the 15% drop-out rate.

### Data collection

Along with the demographic questionnaires, data regarding children’s pain during the venipuncture was rated by the children, their parents and the observer. In addition, saliva cortisol, heart rate(HR) and the percutaneous oxygen saturation (SpO_2_) of children were collected by the staff nurse. Additionally, the venipuncture duration and retaining time of the IV cannulas were also recorded in this study.

Data were collected using the following:

1. Demographic questionnaire;
2. Two pain rating scales;
  a WB for children’s self-evaluation.
  b Revised Face, Legs, Activity, Cry and Consolability Scale (r-FLACC) for parents and observers reporting pain.
3. Human Saliva Cortisol ELISA Kit for collection of children’s saliva.
4. Nellcor OxiMax N65 hand-held pulse oximeter to obtain children’s HR and SpO2.

### Validity and reliability

This study’s design, procedure and writing meet the CONSORT guidelines for randomized controlled trials. The research instruments in this study have high validity and reliability. The primary study outcomes were children’s pain scores measured by the WB during venipuncture procedures. WB and r-FLACC were used in previous research on the Chinese population [15, 16]. Salivary cortisol has been proven a reliable biomarker of acute stress [17–19]. The Cortisol Saliva ELISA kit, which passed the test of the Chinese medical equipment inspection organization, was used to test the saliva cortisol. At least one meeting per month was held to discuss the obstacles and progress during the implementation of the research.

### Ethical considerations

This study was approved by the Children’s Hospital of Fudan university’s Ethical Committee, and written informed consent with assent was obtained from each legal guardian before enrollment. The researchers followed ethical principles of beneficence, respect for human dignity and justice. All the personal information was used anonymously to preserve respondents’ privacy, and no names but unique codes were recorded during this study. All the participants had the right to withdraw from the study at any time.

### Statistical techniques

Data were processed using the Statistical Package for Social Sciences (SPSS Inc., Chicago, IL, USA) for Windows (version 22.0) with statistical significance set to *p* < 0.05. The Mann–Whitney test was used for multiple pairwise comparisons. The Kruskal–Wallis test was used to analyze three or more variables to explore the differences in pain scores and saliva cortisol of children during their venipuncture after the interventions above. In addition, the chi-square test was applied to check the differences in toy preference across ages.

### Intervention

**Fig. 1:**
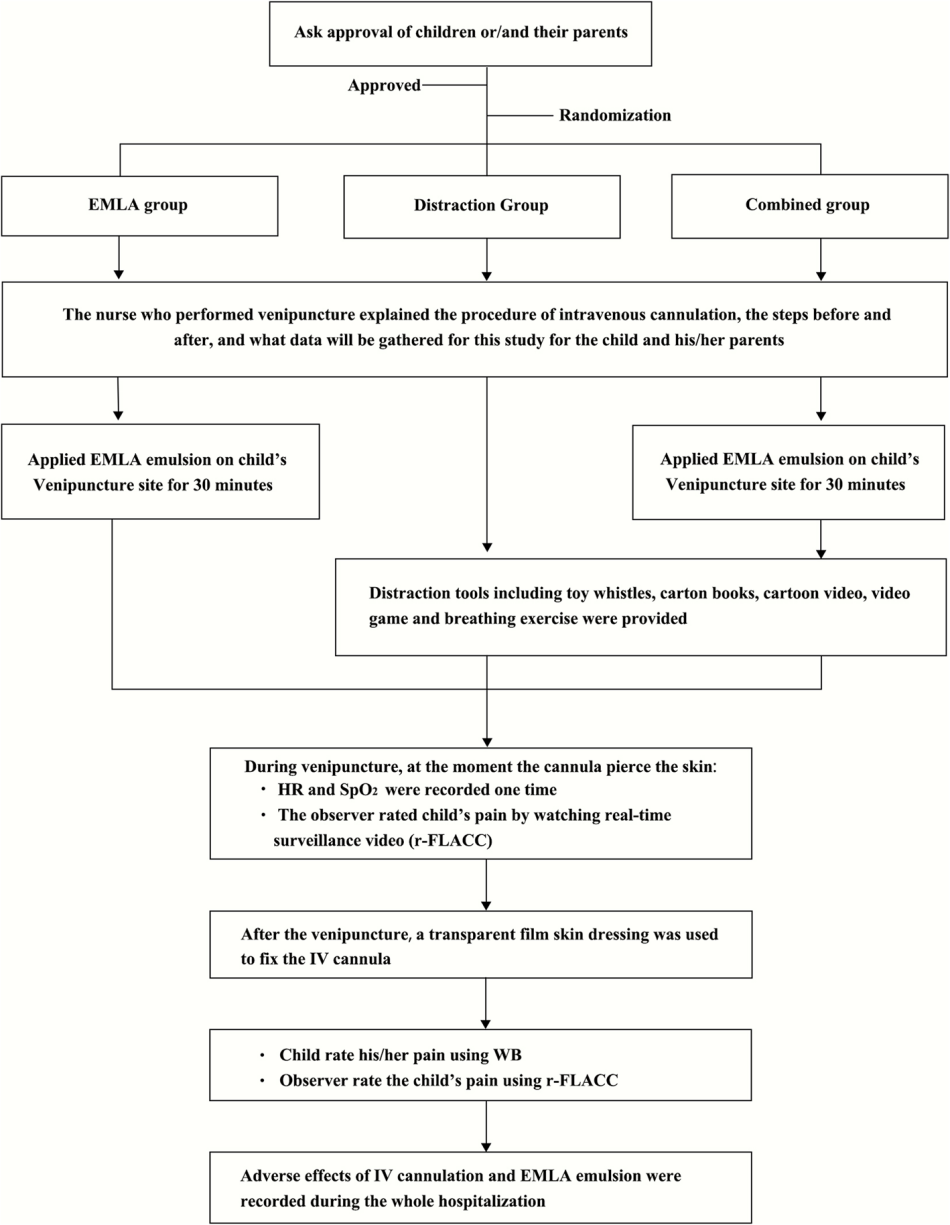
Selection process

The whole procedure of this study is shown in Fig. 1. Hard copies of informed consent were given to all parents. Eligible children were randomly assigned into three groups: EMLA group, distraction group, and combined group. Nurses explained the pain rating scales, pharmacological and non-pharmacological techniques, and the procedure of IV cannulations to children and their company. Every child was held or accompanied by a parent at an upright position throughout the process. At baseline, patients’ age, sex, and ethnicity were recorded before they were grouped.

The nurse performing venipuncture had ten years of experience in the pediatric ward. The IV cannula used was a 19 gauge × 0.7-in. needle and the procedure adhered to standard IV cannulation procedures. The observer-reported pain, HR, and SpO_2_of children for once were defined or collected at the moment the cannula pierced the skin. An observer rated the pain by the r-FLACC scale through real-time surveillance video, which only presents the child’s face and body including the puncture site. Once finished the cannulation, a transparent waterproof adhesive skin dressing was used to fix the cannula. Then saliva samples were taken with a cortisol saliva ELISA assay kit to determine children’s cortisol levels quantitatively. A unified standardized sampling-taking method requires children not to drink, eat and brush their teeth 2 h before the test. All the samples were taken at 8:00–10:00 the morning and 2:00 ~ 4:00 the afternoon [20]. Afterward, the WB and r-FLACC pain scales were given to children and their parents respectively to get self-reported and parents-reported pain. Adverse effects of the IV cannulation or EMLA emulsion were observed and recorded during their entire hospitalization.

For the EMLA group, the specialist nurse who performed IV cannulation determined the puncture site. Next, a thick layer of EMLA emulsion (lidocaine and prilocaine 2.5%/2.5%) was applied on a 1 × 1 cm^2^ area of skin on the cannulation site. The transparent dressing was left to cover the EMLA emulsion for 30 min, then removed and cleaned with a sterile cotton swab. Then nurse performed IV cannulation.

For the distraction group, multiple distraction techniques, including toy whistles, cartoon books, cartoons, and video games were provided for the children to choose from and play with. They were also taught breathing exercises (i.e., inhaling through the nose for 3 s and exhaling for 5 s while they were counting) if they were willing. A play therapist played with the children for 5 min before and throughout the venipuncture procedure.

For the combined group, both EMLA emulsion and distraction techniques were used. First, EMLA emulsion was applied on the pre-puncture site for 30 min to the EMLA group, then 5 min before the venipuncture, the play therapist encouraged them to choose their favorite toys to play with or to practice breathing exercises. During IV cannulation the play therapist also continued distracting the child with toys.

## Results

**Fig. 2:**
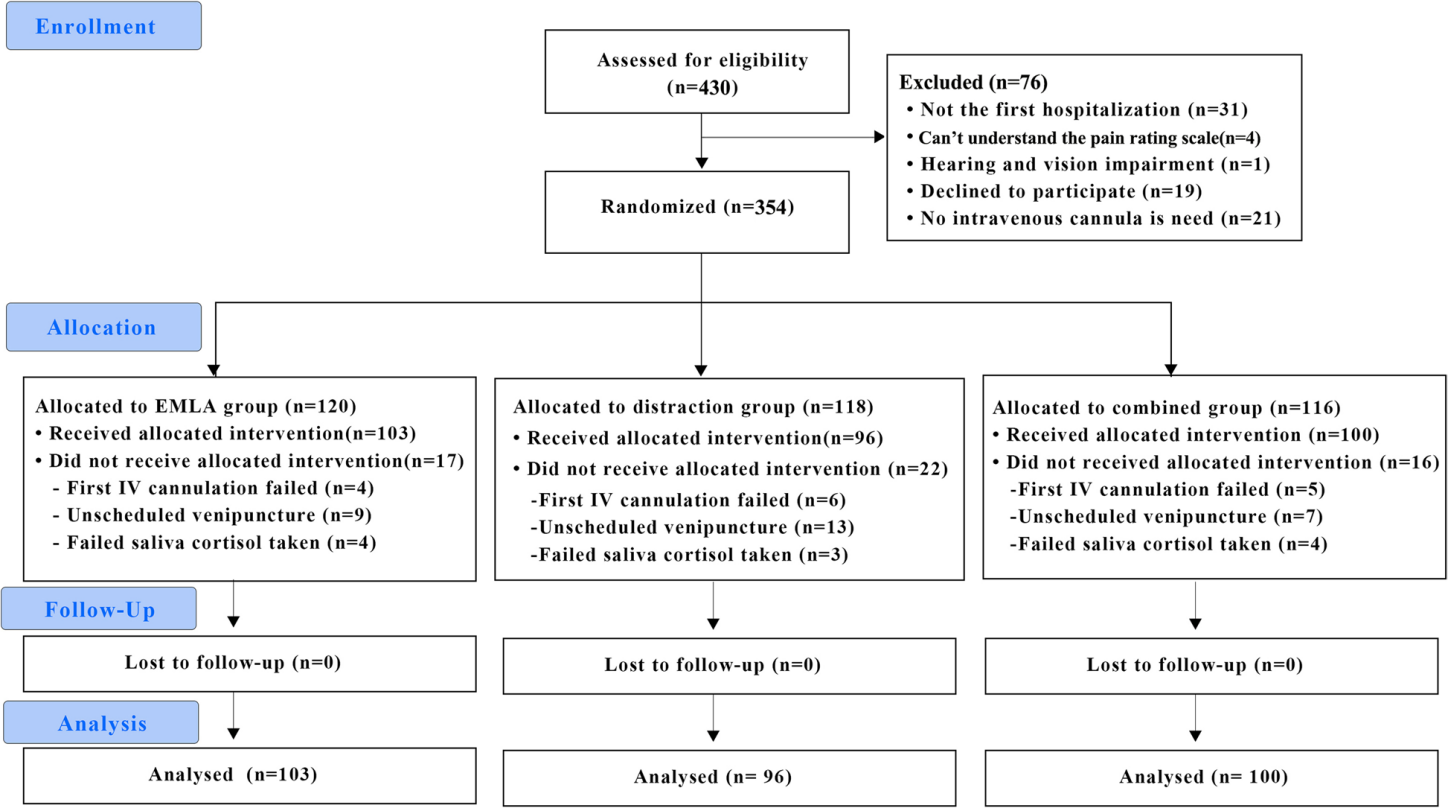
Consort flow diagram

Figure 2 shows the Consort Flow diagram to depict the participant flow of this study. A total of 430 children were admitted to the gastroenterology and respiratory wards from February 19. 2020 to August 10. 2020. After excluding a total of 76 children, which included children who 1) were not in their first hospitalization(*n* = 31); 2) couldn’t understand the pain rating scale(*n* = 4); 3) had a hearing and vision impairment(*n* = 1), 4) declined to participate(*n* = 19) and 5) didn’t need IV cannulation(*n* = 21), 354 eligible children remained. All the participants were randomly allocated to one of three groups which are the EMLA group (*n* = 120), the distraction group (*n* = 118), and the combined group (*n* = 116). After ruling out another 56 children whom 1) had undergone failed first venipuncture (*n* = 15, 4 in the EMLA group, 6 in the distraction group, and 5 in the combined group); 2) were assigned unscheduled venipuncture (*n* = 29, 9 in the EMLA group, 13 in distraction group, and 7 in the combined group) and; 3) failed to take saliva specimen (*n* = 11, 4 in EMLA groups, 4 in distraction group, and 3 in combined group), the final dataset of 103(34.4%) children in the EMLA group, 96(32.2%) children in the distraction group and 100(33.4%) children in the combined group were collected. We considered the venipuncture to be unscheduled if the physician demanded it within 30–45 min, which did not allow enough time for the nurse to apply the EMLA emulsion to patients before inserting the cannula (recommended application time is 60 to 30 min before the procedure) [21, 22], or if there was a need for emergency venipuncture like a blood transfusion.

Table 1 shows no statistical difference in children’s demographic characteristics regarding age(*p* = 0.835), gender(*p* = 0.636), ethnicity(*p* = 0.086) and age range(*p* = 0.516).

**Table 1:** Comparison of groups according to the children’s demographic characteristics

Variable	EMLA group Mean[SD], Min- Max/ N(%)	Distraction group Mean[SD], Min–Max/ N(%)	Combined group Mean[SD], Min- Max/ N(%)	*P*-value
Age(year)	8.4[3.5] 3–16	8.7[3.4] 3–16	8.4[3.8] 3–16	0.835
Gender				0.636
Male	56(54.4)	56(58.3)	55(55.0)	
Female	47(45.6)	40 (41.7)	45(45.0)	
Ethnicity				0.086
Han	100(97)	94(97.9)	98(98)	
Uyghur	2(1.9)	1(1.0)	1(1.0)	
Zhuang	–	1(1.0)	1(1.0)	
Miao	1(0.9)	–	–	
Other	–	–	–	
Age range (year)				0.516
3 ~ 4	40(38.8)	29(30.2)	42(42.0)	
5 ~ 12	47(45.6)	49(51.0)	40(40.0)	
13 ~ 18	16(15.5)	18(18.7)	18(18.0)	

The data of the primary study outcome is presented in Table 2. The median (IQR) pain score of children in all three groups using the WB rating scale is 2(0.00–2.00). The difference in the pain level of the three groups during venipuncture is insignificant (*P* = 0.511). The median (IQR) of pain levels by children’s guardians in the EMLA group, distraction group, and combined group using the r-FLACC Pain Scale are 0.00 (0.00–2.00), 0.00 (0.00–2.00), and 1.00 (0.00–2.00) respectively. The differences in pain levels among the three groups during the venipuncture are not significant (*P* = 0.072). The median (IQR) pain level of the EMLA group, distraction group, and combined group by a nurse using the r-FLACC Pain Scale is 1.00 (0.00–2.00), 1.00 (0.25–2.00), and 1.00 (0.00–2.00) respectively. The *P*-value is 0.909 showing no significant statistical difference between the three group’s pain levels during the venipuncture.

**Table 2:** Comparison of groups according to the children’s self-reported WB, a parent-reported r-FLACC, and Observer reported r-FLACC score

Scale	Reporter	EMLA group Median (IQR)	Distraction group Median (IQR)	Combined Group Median (IQR)	EMLA- Distraction-Combined group (*P*-value)
WB	Self-report	2.00(0.00–2.00)	2.00(0.00–2.00)	2.00(0.00–2.00)	0.051
r-FLACC	Parent report	0.00(0.00–2.00)	0.00(0.00–2.00)	0.00(0.00–2.00)	0.072
r-FLACC	Observer report	1.00(0.00–2.00)	1.00(0.25–2.00)	1.00(0.00–2.00)	0.906

**Fig. 3:**
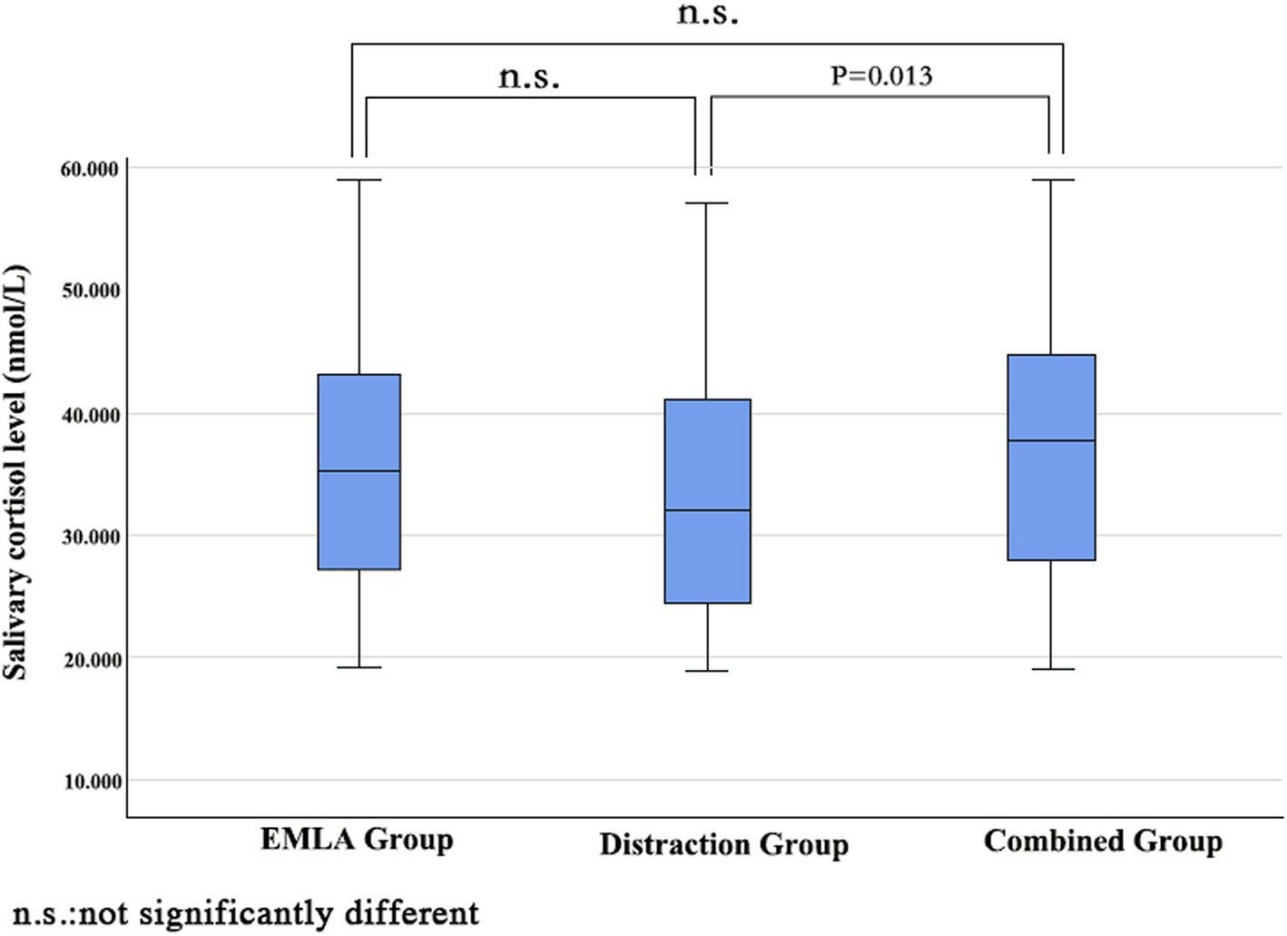
The box plot of three groups in terms of salivary cortisol level

Salivary cortisol levels of the three groups were significantly different [EMLA group: Median(IQR), 35.5(27.1, 43.1); distraction group: 32.0(24.5,41.4); the combined group 37.7(28.1, 44.6), *p* = 0.036]. Children in the distraction group had significantly lower salivary cortisol levels than the combined group (*p* = 0.013)(Fig. 3).

Sympathetic responses like HR and SPO_2_ during venipuncture and time-related metrics such as venipuncture duration and retaining time of IV cannula were also studied (Table 3). The Mean (SD) HR of the EMLA, distraction, and combined groups are 104.02(19.5), 102.49(17.72), and 103.65 (20.17) respectively. There is no significant difference between the three groups (*p* > 0.05). The median (IQR) SPO_2_ of the EMLA, distraction, and combined groups are 98.00(97.00–100.00), 98(97.00–100.00), and 99.00 (97.00–100.00) respectively. There is no significant difference between the three groups (*p* > 0.05).

**Table 3:** Comparison of groups according to the children’s HR, SpO_2_, Venipuncture duration, and retaining time of IV cannulas during their periphery venipuncture

		EMLA group	Distraction group	Combined group	*P*-value
HR	M ± SD	104.02 ± 19.5	102.49 ± 17.72	103.65 ± 20.17	0.844
SpO_2_	Median	99.00	98.00	99.00	0.438
	Interquartile range	97.00–100.00	97.00–100.00	97.00–100.00	
Venipuncture duration (Second, s)	Median	83.00	83.00	85.50	0.440
	Interquartile range	70.00–100.00	79.00–96.00	74.00–98.75	
Retaining time of IV Cannula(Hour, hr)	Median	15.00	15.00	21.00	0.843
	Interquartile range	5.00–47.00	5.00–44.00	5.00–52.00	

For venipuncture duration, the median (IQR) of the EMLA, distraction, and combined groups are 83.00(70.00–100.00)s, 83.00(70.00–96.00)s, and 85.50(74.00–98.75)s. There is no significant difference between the three groups (*p* > 0.05). The median (IQR) retention time of IV cannula of the EMLA, distraction, and combined groups are 15.00(5.00–47.00) hrs, 15.00 (5.00–40.00) hrs, and 21 (5.00–52.00) hrs respectively, and there is no significant difference of three groups (*p* > 0.05).

There is no harm like drug allergy happened to included children when applying the EMLA during the intervention.

## Discussion

This study is one of few comparing the topical anesthetic (i.e. EMLA emulsion), the distraction techniques (i.e. toy whistle, books, cartoon animation, video games, and breathing exercises), and the combination of the two interventions above in mitigating pain during venipuncture in the pediatric ward. Due to ethical considerations, we did not set a control group with no pain control. Instead, results from previous studies were used as a comparison to show that children undergo severe pain when there is no pain relief during venipuncture [5, 23].

Psychological interventions like distractions aim to help children to develop and use their coping skills to manage pain and distress and can be used by non-psychologist [11].

Focusing on something other than pain can keep patients from fear and anxiety [24]. In the distraction group, children’s attention was thus absorbed by the pleasurable objects in a relaxed atmosphere. Since pain is a subjective experience affected heavily by the environment [25], the surroundings where treatment occurs are pivotal. For children, a familiar and pleasant environment eases their vigilance and reduces their anxiety. Providing a window of time for children to choose and engage in the activity of their choice makes a venipuncture room less foreign and hostile. A meta-analysis found that distraction intervention can also relieve the anxiety of parents and nurses(as observers) [11].

In the EMLA group, we used EMLA emulsion to inhibit the transmission of nerve impulses to reduce venipuncture-induced pain. While this method is proven effective in previous studies [9, 21, 25], some drawbacks must be addressed. The optimal application time (60 min) [22] prolongs the procedure, making it unfeasible and impractical in emergencies. However, Hopkins’s study found no correlation between application time and efficacy after 30 min of application [26]. Another study found noticeable pain reduction when EMLA emulsion is applied as short as 5 min before IV cannulation [27]. Other topical anesthetics available, like tetracaine (application time is 30-54 min) and amethocaine (application time is 30 min), is more suitable in busy outpatient settings [28, 29]. Tetracaine is also proven to cause less vasoconstriction and not trigger methemoglobinemia than EMLA [30, 31].

Contrary to expectations, although the results showed the effectiveness of the integration of EMLA emulsion and distraction techniques in the combined group for reducing pain consistent with Heiden’s research, it did not show the statistical differences between the EMLA group and distraction group [32]. The lack of synergistic effect can be attributed to long intervention time and children’s short attention span. The average time of the combined group was 40 min, which consumed children’s attention and caused tiredness. The 5 min of playtime before venipuncture was probably inadequate to overcome 35 min of boredom and irritation, resulting in an effect as if only EMLA emulsion was used.

The salivary collection allows for non-invasive, timed measurement of free cortisol. It is associated with the hypothalamus–pituitary–adrenal axis (HPAA) adaptation to stress. A systematic review shows salivary cortisol is a clear indicator of stress in children and adults [33]. However, we have to consider that variables like circadian rhythm, coping mechanisms, estrogens, or medical conditions et al. could affect cortisol release and HPAA responsivity [17, 34]. Therefore, a standard sample collection method was applied in this study to enhance the validity, rigor, and integrity of the sampling. Parents’ reactions to children’s stress greatly influence children’s ability to deal with stress [35]. An upright position rather than being physically restrained has been shown to increase children’s comfort and decrease pain, preferably with a parent holding up with soothing words [36–38]. In this study, families were encouraged to accompany children in three groups throughout the venipuncture procedure, which greatly supported children and reduced their stress. Plus, with the application of and implementation of the distraction, children’s stress was further decreased. However, the distraction group showed lower salivary cortisol than children in the combined group. This may be due to children in the combined group waiting longer for the venipuncture than children in the distraction group. Sitting in the waiting room before venipuncture has been proven a stressor for children and related to anxiety and pain during venipuncture [39]. In addition, children in the combined group were told the EMLA was for pain relief when the nurse applied it to the skin before the venipuncture. Therefore, that may be taken as a negative implication by children that the procedure is painful and make them stressed before the venipuncture. For HR and SPO_2_, some studies have shown the correlation between pain and heart rate is weak and the relationship between stress and SpO_2_is negligible [40, 41], Therefore, the reliability of HR and SPO_2_ as the indicator of pain and stress response may need more research to prove.

### Limitations

This study has several limitations. Although we successfully blinded the observer and statistician, despite our best efforts, the child and parents can’t be blinded. A placebo could be used identically to the EMLA to blind the participants into three groups. In the distraction group, we considered children’s short attention span and anticipated from previous research that 5 min of playing time was enough to achieve the desired effect [13], however, the optimal duration for children across different ages was not explored. Due to a large number of newly admitted patients every day who need IV cannulation to get therapy as soon as possible in our ward, and the unbalanced nurse-patient ratio, we have to split the difference to find a feasible and practical way to control children’s pain during their IV cannulation and meanwhile meet their therapy needs and hectic clinical work. We shortened the application time of EMLA to 30 min which has been proven effective in controlling pain in our pre-experiment and previous study [26, 27], which intended to make more children be included and benefit from this study. If conditions permit, we suggest an hour application of EMLA to reduce pain further.

## Conclusion

Health workers have an ethical obligation to relieve venipuncture pain and distraction techniques are practical, cost-effective, non-invasive, without side effects, and particularly useful in low-resource settings. At the same time, pharmacological interventions like EMLA is easy-to-use and require minimal training. Medical workers can use the interventions as appropriate for their medical resources and availability meanwhile involves parents whenever possible.  

## Data Availability

The datasets generated and/or analyzed during the current study are not publicly available due to privacy or ethical restrictions. Still, they are available from the corresponding author Prof.Ying Gu on reasonable request.

## Abbreviations

IV: Intravenous
WB: Wong-Baker FACES® Pain Rating Scale
r-FLACC: Revised Face, Legs, Activity, Cry and Consolability Scale
EMLA: A eutectic mixture of local anesthetics
SpO_2_: Percutaneous oxygen saturation

## References

[1] ENA Clinical Practice Guideline Committee, & ENA Board of Directors Liaisons, Methodologist, Staff Liaisons, Administrative Staff. Clinical Practice Guideline: Needle-related or minor procedural pain in pediatric patients. J Emerg Nurs.2019; 45(4). 10.1016/j.jen.2019.05.015 437.e1–437.e32. 10.1016/j.jen.2019.05.015 31280767

[2] Taddio A, Ipp M, Thivakaran S, Jamal A, Parikh C, Smart S, Sovran J, Stephens D, Katz J (2012). Survey of the Prevalence of Immunization Non-Compliance due to Needle Fears in Children and Adults. Vaccine.

[3] McMurtry CM, Pillai Riddell R, Taddio A, Racine N, Asmundson GJ, Noel M, et al. HELPinKids&Adults Team.Far from “just a poke”: Common painful needle procedures and the development of needle fear. Clin J Pain. 2015;31:S3–11. 10.1097/AJP.0000000000000272 PMC4900413 26352920

[4] Noel M, McMurtry CM, Chambers CT, McGrath PJ (2010). Children's memory for painful procedures: the relationship of pain intensity, anxiety, and adult behaviors to subsequent recall. J Pediatr Psychol.

[5] Dalvandi A, Ranjbar H, Hatamizadeh M, Rahgoi A, Bernstein C (2017). Comparing the effectiveness of vapor coolant spray and lidocaine/procaine cream in reducing the pain of intravenous cannulation: A randomized clinical trial. Am J Emerg Med.

[6] Sikorova L, Hrazdilova P (2011). The effect of psychological intervention on perceived pain in children undergoing venipuncture[J]. Biomed Pap Med Fac Univ Palacky Olomouc Czech Repub.

[7] Baxter AL, Ewing PH, Young GB, Ware A, Evans N, Manworren RC (2013). EMLA application exceeding two hours improves pediatric emergency department venipuncture success. Adv Emerg Nurs J.

[8] Lander JA, Weltman BJ, So SS. EMLA and amethocaine for reduction of children’s pain associated with needle insertion. Cochrane Database Syst Rev. 2006;(3):CD004236. 10.1002/14651858.CD004236.pub2 16856039

[9] Fetzer SJ (2002). Reducing venipuncture and intravenous insertion pain with eutectic mixture of local anesthetic: a meta-analysis. Nurs Res.

[10] Lunoe MM, Drendel AL, Levas MN (2015). A Randomized Clinical Trial of Jet-Injected Lidocaine to Reduce Venipuncture Pain for Young Children. Ann Emerg Med.

[11] Birnie KA, Noel M, Chambers CT, Uman LS, Parker JA. Psychological interventions for needle-related procedural pain and distress in children and adolescents. Cochrane Database Syst Rev. 2018;10(10):CD005179. Published 2018 Oct 4. 10.1002/14651858.CD005179.pub4. 10.1002/14651858.CD005179.pub4 PMC6517234 30284240

[12] Uman LS, Chambers CT, McGrath PJ, Kisely S (2008). A systematic review of randomized controlled trials examining psychological interventions for needle-related procedural pain and distress in children and adolescents: an abbreviated Cochrane review. J Pediatr Psychol.

[13] Gold JI, Kim SH, Kant AJ, Joseph MH, Rizzo AS. Effectiveness of virtual reality for pediatric pain distraction during i.v. placement. Cyberpsychol Behav. 2006;9(2):207–12. 10.1089/cpb.2006.9.207. 10.1089/cpb.2006.9.207 16640481

[14] Gershon J, Zimand E, Lemos R, Rothbaum BO, Hodges L (2003). Use of virtual reality as a distractor for painful procedures in a patient with pediatric cancer: a case study. Cyberpsychol Behav.

[15] Bai J, Hsu L, Tang Y, van Dijk M (2012). Validation of the COMFORT Behavior scale and the FLACC scale for pain assessment in Chinese children after cardiac surgery. Pain Manag Nurs.

[16] Gong ZR, Shu M, Wang CM, Zhu Y& Luo SH. Validation of Wong-Baker FACES Rating Scale in comfort assessment among children with acute fever and aged 0–5 years[J]. Chinese Journal of Evidence -Based Pediatric, 2015, 10(6): 401–404.

[17] Hellhammer DH, Wüst S, Kudielka BM (2009). Salivary cortisol as a biomarker in stress research. Psychoneuroendocrinology.

[18] Kirschbaum C, Hellhammer DH (1994). Salivary cortisol in psychoneuroendocrine research: recent developments and applications. Psychoneuroendocrinology.

[19] Patil SJ, Shah PP, Patil JA, Shigli A, Patil AT, Tamagond SB (2015). Assessment of the changes in the stress-related salivary cortisol levels to the various dental procedures in children. J Indian Soc Pedod Prev Dent.

[20] Salimetrics LLC, SalivaBio LLC. Saliva collection and handling advice. 2011. Available from: http://www.salimetrics.com. Accessed December 1, 2019.

[21] Matsumoto T, Chaki T, Hirata N, Yamakage M. The eutectic mixture local anesthetics (EMLA) cream is more effective on venipuncture pain compared with lidocaine tape in the same patients. JA Clin Rep. 2018;4(1):73. Published 2018 Oct 8. 10.1186/s40981-018-0210-1 10.1186/s40981-018-0210-1 PMC6966927 32026028

[22] Lycka BAEMLA (1992). A new and effective topical anesthetic. J Dermatol Surg Oncol.

[23] Sikorova L, Hrazdilova P (2011). The effect of psychological intervention on perceived pain in children undergoing venipuncture. Biomed Pap Med Fac Univ Palacky Olomouc Czech Repub.

[24] Zengin S, Kabul S, Al B, Sarcan S, Dogan M, Yildirim C (2013). Effects of music therapy on pain and anxiety in patients undergoing port catheter placement procedure. Complement Ther Med.

[25] Rogers TL, Ostrow CL (2004). The use of EMLA cream to decrease venipuncture pain in children. J Pediatr Nurs.

[26] Hopkins CS, Buckley CJ, Bush GH.Pain-free injection in infants. Use of a lignocaine-prilocaine cream to prevent pain at intravenous induction of general anaesthesia in 1–5-year-old children. Anaesthesia. 1988;43:198–201. 3284402

[27] Smith MS, Holder PG, Leonard K. Efficacy of a five-minute application of EMLA cream for the management of pain associated with intravenous cannulation. Internet Journal of Anesthesiology. 2001; 6(1).

[28] Szmuk P, Szmuk E, Ezri T (2005). Use of needle-free injection systems to alleviate needle phobia and pain at injection. Expert Rev Pharmacoecon Outcomes Res.

[29] Molodecka J, Stenhouse C, Jones JM, Tomlinson A (1994). Comparison of percutaneous anaesthesia for venous cannulation after topical application of either amethocaine or EMLA cream. Br J Anaesth.

[30] Nott MR (2001). EMLA or Ametop, and for how long?. Anaesthesia.

[31] Choy L, Collier J, Watson AR (1999). Comparison of lignocaine-prilocaine cream and amethocaine gel for local analgesia before venepuncture in children. Acta Paediatr.

[32] Hedén LE, von Essen L, Ljungman G (2011). Effect of morphine in needle procedures in children with cancer. Eur J Pain.

[33] Aguilar Cordero MJ, Sánchez López AM, Mur Villar N, García García I, Rodríguez López MA, Ortegón Piñero A, Cortés Castell E. Cortisol salival como indicador de estrés fisiológico en niños y adultos; revisión sistemática [Salivary cortisol as an indicator of physological stress in children and adults; a systematic review]. Nutr Hosp. 2014 May 1;29(5):960–8. Spanish. 10.3305/nh.2014.29.5.7273. PMID: 24951973. 10.3305/nh.2014.29.5.7273 24951973

[34] Wu JY, Hsu SC, Ku SC, Ho CC, Yu CJ, Yang PC (2008). Adrenal insufficiency in prolonged critical illness. Crit Care.

[35] Sparks LA, Setlik J, Luhman J (2007). Parental holding and positioning to decrease IV distress in young children: A randomized controlled trial. J Pediatr Nurs.

[36] Wente SJ (2013). Nonpharmacologic pediatric pain management in emergency departments: A systematic review of the literature. J Emerg Nurs.

[37] Taddio A, Shah V, McMurtry CM, et al.; HELPinKids&Adults Team. Procedural and physical interventions for vaccine injections: Systematic review of randomized controlled trials and quasi-randomized controlled trials. Clin J Pain. 2015;31 (10 Suppl):S20–37. 10.1097/AJP.0000000000000264 PMC4900423 26352919

[38] Kaluza AJ, Aydin AL, Cordes BL, et al. A Sorrow Shared Is a Sorrow Halved? Patient and Parental Anxiety Associated with Venipuncture in Children before and after Liver Transplantation. Children (Basel). 2021;8(8):691. Published 2021 Aug 11. 10.3390/children8080691. 10.3390/children8080691 PMC8394744 34438582

[39] Bossart P, Fosnocht, D, SwansonE. Changes in heart rate do not correlate with changes in pain intensity in emergency department patients. The Journal of emergency medicine.2007; 32(1): 19–22. 10.1016/j.jemermed.2006.05.029. 10.1016/j.jemermed.2006.05.029 17239728

[40] GÜMÜŞ D, TOPAL F, KÖŞGEROĞLU N. The Effect of Academic Stress on Oxygen Saturation in Paramedic Students. International symposium on innovative approaches in scientific studies.2018; 2: 250–250. 10.36287/setsci ISSN: 2687–5527.

[41] Gershon J, Zimand E, Lemos R, Rothbaum BO, Hodges L (2003). Use of virtual reality as a distractor for painful procedures in a patient with pediatric cancer: a case study. Cyberpsychol Behav.

